# Inositol hexaphosphate modulates the behavior of macrophages through alteration of gene expression involved in pathways of pro‐ and anti‐inflammatory responses, and resolution of inflammation pathways

**DOI:** 10.1002/fsn3.2286

**Published:** 2021-04-10

**Authors:** Yinshen Wee, Chieh‐Hsiang Yang, Shau‐Kwaun Chen, Yu‐Chun Yen, Ching‐Shuen Wang

**Affiliations:** ^1^ Department of Pathology University of Utah Salt Lake City UT USA; ^2^ Huntsman Cancer Institute University of Utah Salt Lake City UT USA; ^3^ Institute of Neuroscience National Chengchi University Taipei Taiwan; ^4^ Biostatistics Center Office of Data Science Taipei Medical University Taipei Taiwan; ^5^ School of Dentistry College of Oral Medicine Taipei Medical University Taipei Taiwan

**Keywords:** anti‐inflammatory, dietary supplement, formyl peptide receptor 2, Inositol hexaphosphate, lipoxygenases, M2a macrophages, macrophage polarization, phytic acid, resolution of inflammation, tissue repair

## Abstract

Inositol hexaphosphate (IP6) is a dietary compound commonly obtained from corn, rice, etc. Although we may consume significant amount of IP6 daily, it is unclear whether this diet will impact macrophages’ fate and function. Therefore, we characterized the underlying relationship between IP6 and macrophage polarization in this study. We specifically examined the signature gene expression profiles associated with pro‐ and anti‐inflammatory responses, and resolution of inflammation pathways in macrophages under the influence of IP6. Interestingly, our data suggested that IP6 polarizes bone marrow‐derived macrophages (BMDM) into an M2a‐like subtype. Our results also demonstrated that IP6 reduces lipopolysaccharide‐induced apoptosis and pro‐inflammatory responses in macrophages. In contrast, the expression levels of genes related to anti‐inflammatory responses and resolution of inflammation pathways are upregulated. Our findings collectively demonstrated that IP6 has profound modulation effects on macrophages, which warrant further research on the therapeutic benefits of IP6 for inflammatory diseases.

## Graphical abstract

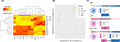

## 1 INTRODUCTION

Immune cells require nutrients to maintain their health and functions (Conlon & Bird, 2015; McMacken & Shah, 2017; Singh et al., 2017). The evidence suggests that dietary compounds can modulate the functions of immune cells directly or indirectly (Conlon & Bird, 2015; McMacken & Shah, 2017; Singh et al., 2017). For example, dietary components such as curcumin help mitigate experimental autoimmune myocarditis (EAM) by directly promoting M2 macrophage polarization (Gao et al., 2015), while eicosapentaenoic acid (EPA) alters host immune cell function indirectly through the production of various kinds of metabolites. Alterations of those functions are the consequences of the interaction between EPA and the gut commensal bacteria (Chapkin et al., 2009; Narayan et al., 2006; Zhuang et al., 2020). Our findings highlight the importance of dietary compounds in maintaining an effective immune system. Therefore, dietary modulation of the inflammatory reaction in inflammatory diseases, including but not limited to diabetes, inflammatory cardiovascular diseases, and cancer, is beginning to be appreciated (Chang et al., 2020; Dugo et al., 2017; Hu, 2003; Kwon, 2020; McMacken & Shah, 2017; Prevete et al., 2018; Shamsuddin, 2002; Shin et al., 2020).

Inositol hexaphosphate (IP6, also known as phytic acid) is the primary source for the storage of phosphate and inositol in edible plant seeds, legumes, grains, and most mammalian tissues (García‐Estepa et al., 1999; Graf et al., 1987). IP6 has been applied as a dietary intervention for treating inflammatory disorders in several experimental models (Da Silva et al., 2019; del Mar Arriero et al., 2012a; Grases et al., 2006; Kumar et al., 2004; Lv et al., 2015; Shin et al., 2020). A growing body of evidence suggests that IP6 has beneficial effects on suppressing the progression of diseases such as inflammatory bowels, diabetes, inflammatory cardiovascular disorders, and malignant tumors (Bizzarri et al., 2020; Grases et al., 2006; Liao et al., 2007; Shamsuddin, 2002; Silva & Bracarense, 2016). In addition, previous studies have demonstrated that IP6 has a profound impact on the formation of chelating free oxygen radicals, thereby inhibiting lipid peroxidation and alleviating exacerbation of pro‐inflammatory‐related diseases (Graf & Eaton, 1990; Katayama, 1999; Ko & Godin, 1990; Lv et al., 2015; Shin et al., 2020; Tan & Norhaizan, 2020). These medical benefits outweigh the anti‐nutritional side effects of IP6; regardless, IP6 is also an anti‐nutritional component that has been reported to hinder the absorption of ions.

With respect to the effects of IP6 on cellular functions, IP6 has been shown to enhance the function of natural killer cells and regulate the expression of pro‐inflammatory cytokines TNFα and IL‐1β in neutrophils (Zhang et al., 2005). IP6 also attenuates the inflammation‐induced apoptosis of neurons, indicating its potential for treating neurodegenerative diseases (López‐Gambero et al., 2020; Xu et al., 2008, 2011). Multiple lines of evidence suggest an essential biological role for IP6. However, some facts require further investigation, especially the role of IP6 in modulating immune cells and associated inflammatory responses. Macrophages contribute to various exacerbated inflammatory diseases, including diabetes, atherosclerosis, rheumatoid arthritis, obesity, and cancer (Chawla et al., 2011; Fujiwara & Kobayashi, 2005; Omoruyi et al., 2020; Özturan et al., 2019). However, little is known about the effect of IP6 on macrophages.

It is now well‐accepted that macrophages can be differentiated into two distinct subtypes, M1 and M2, in response to stimuli from the microenvironment (Biswas et al., 2012). The classic M1 macrophages predominately produce pro‐inflammatory cytokines, such as interleukin‐6 (IL‐6) and tumor necrosis factor α (TNFα), protecting against invading pathogens. In contrast, the alternatively activated M2 macrophage produces anti‐inflammatory cytokines, such as interleukin‐10 (IL‐10) and arginase‐1 (Arg‐1), promoting tissue repair and remodeling (Biswas et al., 2012; Liu et al., 2014; Wang et al., 2019). In recognition of macrophage plasticity in adapting to a highly dynamic microenvironment, recent studies have been undertaken primarily to identify and design a nutraceutical‐based diet or supplements on the modulation of macrophage functions (Chang et al., 2020; Dugo et al., 2017; Gao et al., 2015; Titos et al., 2011). Indeed, the functions of IP6 make it one of the emerging classes of dietary compounds that have been linked to the prevention of inflammatory diseases. However, it is not clear whether IP6 will affect the fate and function of macrophages. Therefore, this study aims to investigate the polarization of macrophages and the resolution of inflammation by monitoring the changes in gene expression profiles under the influence of IP6.

## 2 MATERIALS AND METHODS

### 2.1 Reagents

Inositol hexaphosphoric acid (IP6, #P8810) and Lipopolysaccharides from *E. coli* 0111:B4 (LPS, #L2630) were purchased from Sigma‐Aldrich (St. Louis, MO, USA). IP6 powder was dissolved in distilled water at 20 mg/ml with pH adjusted to 7.4. The stock solution was then stored at −20°C before being further diluted for cell culture. LPS was prepared in a phosphate buffer at 1 mg/ml and stored at −20°C before use. Dulbecco's Modified Eagle Medium (DMEM, #12430047), RPMI1640 (#A4192301), phosphate‐buffered saline solution (PBS, #70011), and fetal bovine serum (FBS, #26140), L‐glutamine (#21051024), penicillin–streptomycin (#15140148), paraformaldehyde (#R37814), and Trypsin‐EDTA solution (#R001100) were purchased from Thermo Fisher Scientific (Carlsbad, CA, USA).

### 2.2 Isolation and culture of bone marrow‐derived macrophages (BMDM)

Fresh bone marrow‐derived macrophages were prepared as previously described (Wee et al., 2012, 2015), using an L929 conditioned medium. Briefly, L929 cells purchased from Sigma‐Aldrich (St. Louis, MO, USA, #85103115) were cultured for 4 days and L929 supernatant was collected and stored at −20°C before use. BMDM cells were harvested by flushing bone marrow cells from femurs and tibia of mice, removing red blood cells using RBC lysis buffer and resuspending cells in RPMI1640 supplemented with 20% FBS, 30% L929 supernatant, 100 U/ml penicillin, 100 µg/ml streptomycin, and 2 mM L‐glutamine. Cells were then cultured in petri dishes and incubated at 37°C in a 5% CO2 atmosphere. After four days of culture, a fresh medium was added and incubated for an additional three days. After seven days of culturing, supernatants were removed, and cells were washed with sterile PBS. The cells were then centrifuged at 200xg for five minutes and resuspended in RPMI 1,640. Cells were then cultured for 12 hr before treatment. The treatment of BMDM was 200 µM for IP6 and 100 ng/ml for LPS at 37°C for 24 hr. Another macrophage cell line J774A.1 was purchased from Bioresource Collection and Research Center (Hsinchu, Taiwan, # 60,140). J774A.1 cells were maintained and cultured in DMEM with 4 mM L‐glutamine, 1.5 g/L sodium bicarbonate, 4.5 g/L glucose, and 10% FBS in a 100 mm plastic dish at 37°C in a CO_2_ incubator (5% CO_2_‐95% humidified air). The cells were passed every 3 days and maintained before experiments.

### 2.3 Immunostaining of Ki67

Cells were fixed with 4% paraformaldehyde and permeabilized using 0.1% Triton X‐100 at room temperature for 10 min, the cell pellets were then washed and resuspended in 2% bovine serum albumin (Thermo Fisher Scientific, Carlsbad, CA, USA, # A2153) with Ki‐67 antibody (Abcam, #ab16667) at 1:200 dilutions. Subsequently, the cells were washed with PBS and incubated with goat anti‐rabbit Alexa488 secondary antibody (Abcam, #ab150077) at 1:500 dilutions. Cells were counterstained with Phalloidin‐iFluor 488 Reagent (Abcam, # ab176753) and DAPI solution (Thermo Fisher Scientific, #62248) at RT for 30 min. Cells were mounted on coverslips and immunofluorescence images of Ki67 stained cells were captured at 20× magnification using Leica TCS SP5. The number of Ki67 positive cells was counted using ImageJ software.

### 2.4 Quantitative polymerase chain reaction

Treated BMDM from each group was harvested and immersed in an RNA stabilization solution (Themo Fisher Scientific, Carlsbad, CA, USA, #AM7020) at 4°C overnight. The RNA stabilization solution was removed on the next day, and total RNAs were extracted using the RNAeasy Kit (QIAGEN, Hilden, Germany, #74104) according to the manufacturer's instructions. One µg of total RNAs was reverse transcribed into 20 µl cDNAs with the iScript Reverse Transcription Kit according to the manufacturer's instructions (Bio‐Rad, Hercules, CA, USA, #1708840). The reactions were carried out by adding the following reagents: 1 μM of each primer (stock was prepared at 10 μM; see Table S1), 25 ng cDNA, and 10 μL of 2 × SYBR Green master mixes (Bio‐Rad, Hercules, CA, USA, #1725121) in a total of 25 μl. Polymer Chain Reaction was performed on 96 well plates at the following temperature cycles: Step 1:95°C for 5 min; Step 2:95°C for 30 s, 60°C for 30 s, and 72°C for 35 s for 35 more cycles; Step 3:72°C for 5 min. Relative fold changes of gene expression were normalized using β‐actin, and results were plotted and analyzed using Prism software (GraphPad Software Inc.).

### 2.5 Bioinformatic analyses

A principal component analysis (PCA) of the gene expression levels between PBS, LPS, IP6, and IP6+LPS in BMDM was quantified using qPCR. The statistical analysis used a logarithmic (log2) transformation of the data to stabilize the variance. The mean values of triplicate qPCR assays for each sample were analyzed statistically using the prcomp function in R (www.r‐project.org). The PCA results are shown as the two‐dimensional contribution scores for component numbers 1 and 2 (PC1 and PC2). The contribution scores were produced by conversion from each eigenvector value, with 11 genes.

### 2.6 Statistical analysis

Data were mean ± *SD* of the results from three or more experiments. *p* < .05 were calculated from a two‐tailed *t* test or a two‐way analysis of variance (ANOVA) with Prism (GraphPad Software Inc.) and taken to represent significant differences.

## 3 RESULTS

### 3.1 IP6 does not affect the proliferation of nonactivated murine macrophages

**FIGURE 1:**
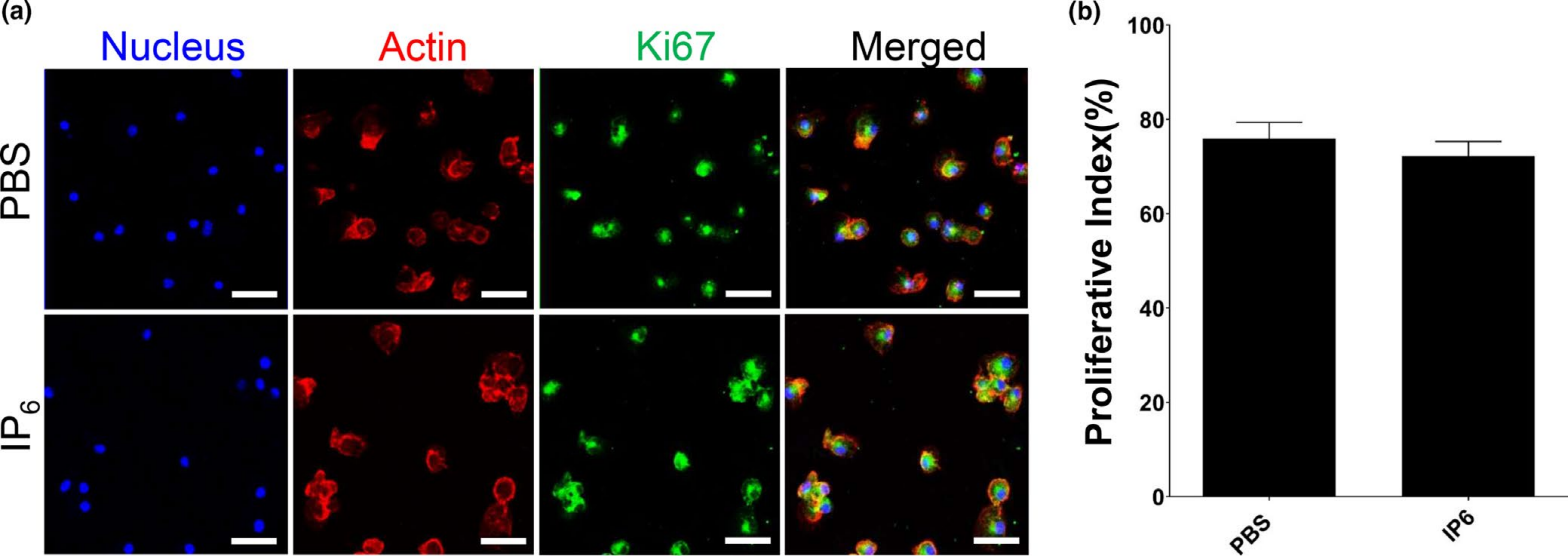
Treatment with IP6 does not affect the proliferation in nonactivated BMDM. (a) Immunofluorescence staining of the proliferation marker Ki67 in each group: Actin (red), Ki67 (green), and DNA (blue). The results from three independent experiments and representative images are shown. Scale bars = 50 μm. (b) Statistical analysis of Ki‐67‐positive cells in each group

IP6 at a concentration lower than 100 µM has been shown to be non‐toxic to various cell types, including murine macrophage RAW264.7 (derived from Balb/c mice) (Anekonda et al., 2011; del Mar Arriero et al., 2012; Masunaga et al., 2019). Little is known about the cytotoxic effect of IP6 on nonactivated primary immune cells, such as primary bone marrow‐derived macrophages (BMDM). Here, we used 200 µM IP6 to investigate the effects of IP6 on the proliferation of nonactivated BMDM. This concentration of IP6 was two times more concentrated than the highest concentration that tested in the aforementioned study (del Mar Arriero et al., 2012). In addition, the concentrations of IP6 at more than 200 µM have been demonstrated as the concentrations that are required to activate mononuclear cells for TNFα production in the presence of LPS from various bacteria (Weglarz et al., 2008). TNFα plays an important role in triggering cell death and tissue damage in many different cell types (Gaur & Aggarwal, 2003). This study also further showed that increasing the concentration of IP6 to 500 µM or 1,000 µM did not cause a significant increase in TNFα production as compared to 200 µM IP6 (Weglarz et al., 2008). Antibodies against Ki‐67 protein have been widely used as proliferation markers for the detection of dividing cells. Therefore, a strong/positive Ki67 staining in the cell nuclei suggested that the cells were dividing into new cells. Our results demonstrated that BMDM treated with IP6 has a similar number of Ki67‐positive cells as cells treated with PBS only, suggesting that IP6 does not affect the proliferation of nonactivated murine macrophages (Figure 1a,b). We further examined whether IP6 has the same effect on murine macrophage cell line J774A.1 using the same concentration of IP6 that was used for BMDM (primary cells). Similar results were observed in J774A.1 cells, showing that IP6 at 200 µM does not affect cell proliferation of J774A.1 cells (Figure S1).

### 3.2 IP6 significantly polarizes M0 BMDM into an M2a‐like subtype

**FIGURE 2:**
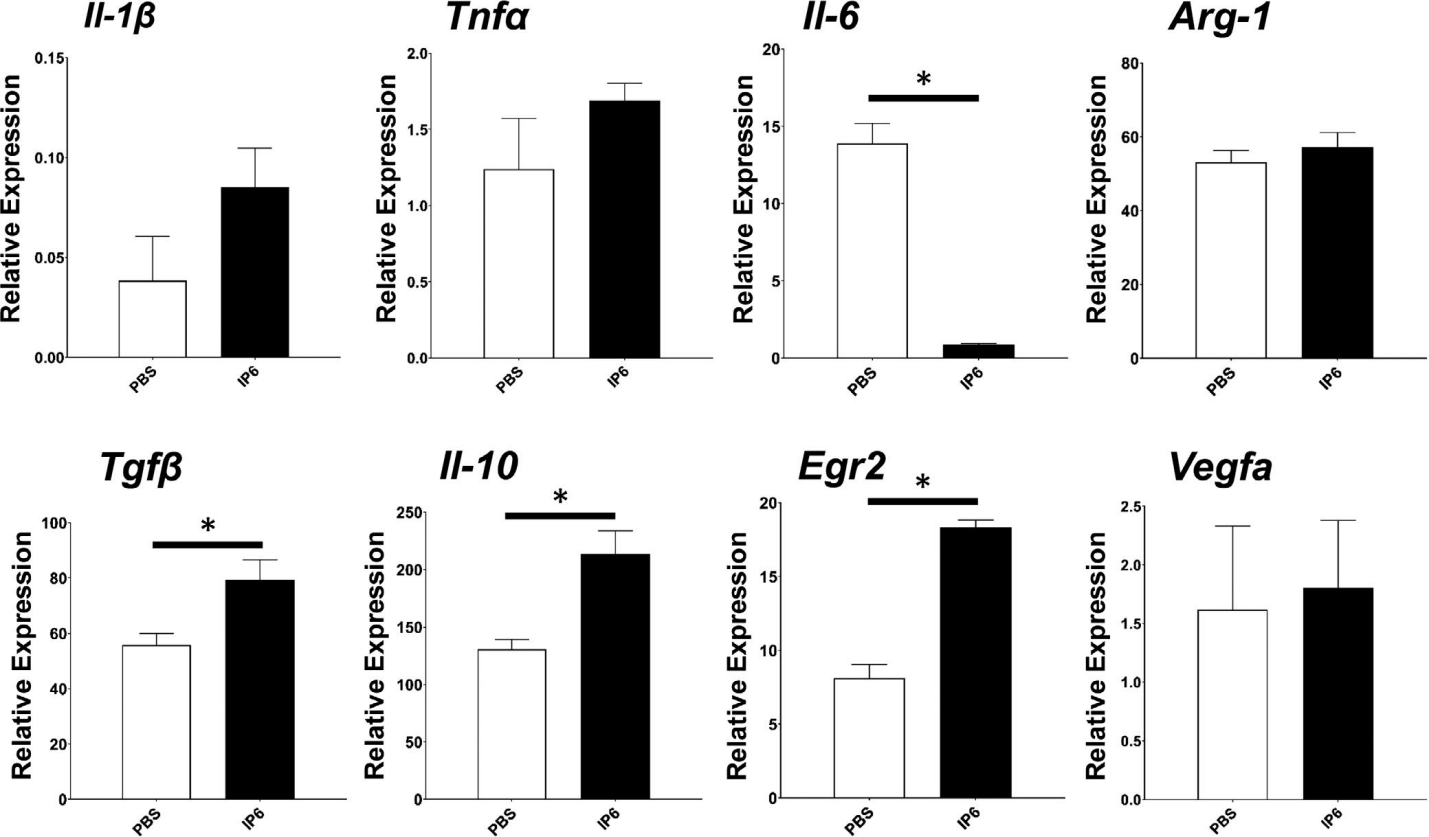
Treatment with IP6 promotes M2a‐like BMDM polarization. qPCR analysis of M1 or M2 markers in BMDMs treated with or without IP6 for 24 hr. The results from three independent experiments are presented as the mean ± *SD*. *p < .05

Macrophages are highly plastic and can respond to diverse stimuli. Therefore, we sought to understand whether IP6 has an impact on modulating the plasticity of macrophages. To test this, we treated BMDM with 200 μM IP6 and measured gene expression signatures related to M1/M2 macrophage polarization. Egr2 has been demonstrated as an essential marker in M2a‐like macrophages (Jablonski et al., 2015; Lu et al., 2013), which play critical roles in promoting cell growth and tissue repairing. Our results showed that IP6 polarizes M0 BMDM into an M2a‐like subtype by significantly increasing the gene expression level of *Egr2* and other M2a‐like macrophage signature genes such as *Tgfβ* and *Il‐10* (Figure 2). In contrast, the expression of genes relevant to M1 macrophages was either reduced (*Il‐6*) or was not altered (*Tnfα* and *Il‐1β*) in response to IP6 stimulation.

### 3.3 IP6 alleviates LPS‐induced cytotoxicity in murine macrophages and suppresses pro‐inflammatory gene expression

**FIGURE 3:**
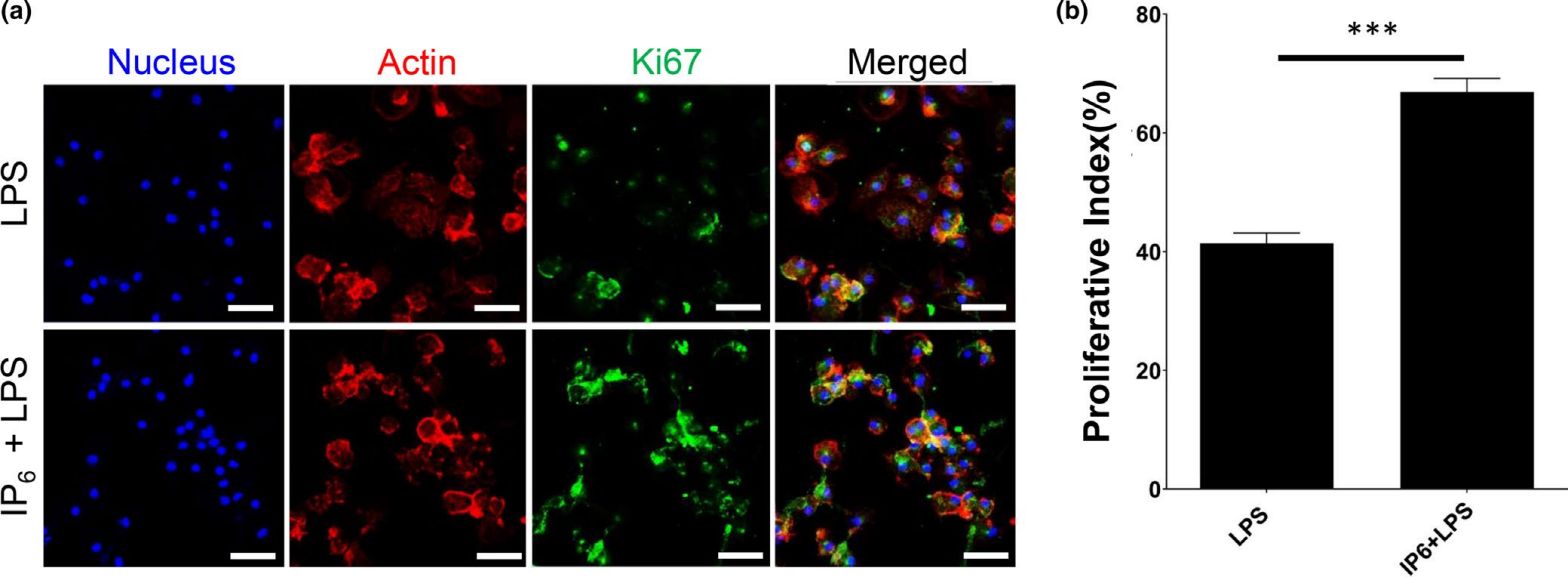
Treatment with IP6 significantly prevents LPS‐mediated cell cycle arrest in BMDM. (a) Immunofluorescence staining of the proliferation marker Ki67 in each group: Actin (red), Ki67 (green), and DNA (blue). The results from three independent experiments and representative images are shown. Scale bars = 50 μm. (b) Statistical analysis of Ki‐67‐positive cells in each group. ***p < .001

**FIGURE 4:**
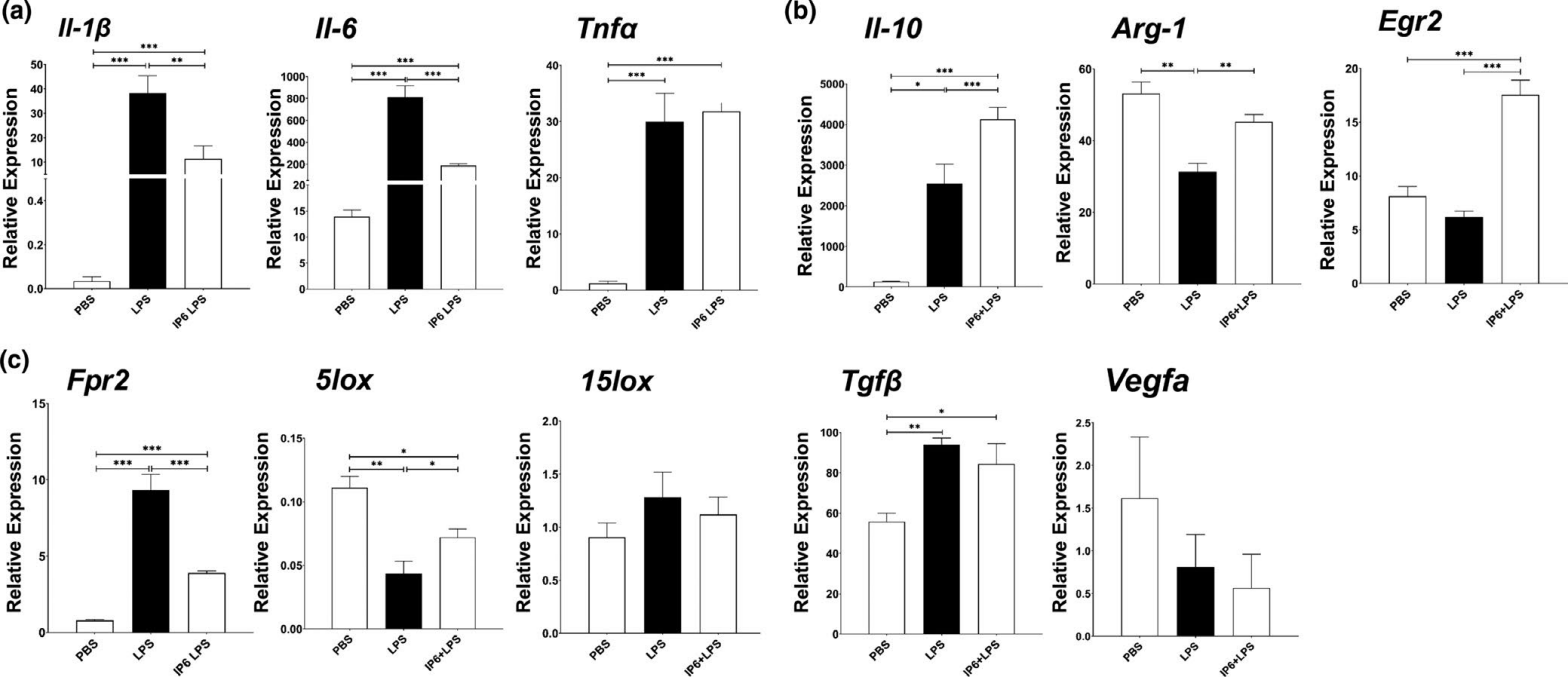
Treatment with IP6 alters LPS‐mediated gene expression in BMDM. qPCR analysis of (a) pro‐inflammatory cytokines, (b) anti‐inflammatory cytokines, and (c) resolution of inflammation in BMDMs treated with or without IP6 in the presence of LPS stimulation for 24 hr. The results from three independent experiments are presented as the mean ± *SD*. *p < .05, **p < .01, and ***p < .001

LPS is a potent activator of macrophages. To understand the cytotoxicity effects of IP6 on activated macrophages, we treated the BMDM with 100 ng/ml of LPS. Our results suggested that exposure of BMDM to the addition of IP6 significantly alleviates LPS‐reduced cell proliferation (Figure 3a,b). In summary, a comparison of BMDM treated with LPS alone to a combination of IP6 and LPS revealed combined treatment of IP6 and LPS resulted in an elevated Ki‐67 proliferative index (70%). In comparison, the LPS‐treated BMDM had a proliferative index of 40% (Figure 3b). Our data also showed that BMDM treated with IP6 significantly suppresses gene expression of LPS‐induced pro‐inflammatory cytokine *Il‐1β* and *Il‐6*, whereas *Tnfα* remained unchanged compared to LPS alone (Figure 4a). Our results indicated that IP6 significantly alleviates LPS‐reduced cell proliferation and gene expression associated with pro‐inflammatory cytokine production.

### 3.4 The effect of IP6 on the modulation of anti‐inflammatory responses and resolution of inflammation‐associated gene expression in murine macrophages

After demonstrating that IP6 reduces the cytotoxic effect and suppresses pro‐inflammatory gene expression in response to LPS stimulation, we further examined whether IP6 affects the modulation of anti‐inflammatory gene expression in LPS‐induced M1‐like phenotypes (LPS‐treated BMDM). The expression levels of *Il‐10*, *Arg‐1*, and *Egr2* increased when IP6 was exposed to LPS‐treated BMDM (LPS + IP6), compared with BMDM treated with LPS only. Other genes, such as *Tgfβ* and *Vegfa*, were unaffected (Figure 4b).

Previous studies suggested that G‐protein‐coupled receptor Fpr2 modulates anti‐inflammatory responses and activates the resolution of inflammation critical to tissue homeostasis (Chen et al., 2013; Corminboeuf & Leroy, 2015, p. 2; Sansbury et al., 2020, p. 2; Vital et al., 2016). Enzymes such as 5lox and 15lox are shown to be essential for pro‐resolving pathways (Buckley et al., 2013; Serhan & Savill, 2005). Deletion of either of these two enzymes resulted in modulation of macrophage polarization toward an M1 phenotype (Lasky et al., 2015). Here, we examined whether IP6 alters gene expression of *Fpr2, 5lox*, and *15lox*; these are critical for the resolution of inflammation. Our results demonstrated that LPS significantly increased the *Fpr2* gene expression and reduced the *5lox* gene expression in BMDM. These findings are not surprising because LPS is a potent inducer of inflammation. As a result, the gene expression of *Fpr2* is upregulated to counterbalance the exacerbated inflammatory responses caused by LPS.

In contrast, we found that IP6 reduced the overexpression of *Fpr2* when LPS‐treated BMDM was exposed to IP6, compared to BMDM treated with LPS only (Figure 4c). We also found that IP6 increased *5lox* expression in BMDM treated with LPS, compared to BMDM treated only with LPS while leaving the *15lox* unaffected. Together with our findings on the anti‐inflammatory function of IP6 (e.g., upregulation of *Il‐10,* *Arg‐1*, and *Egr2*), we concluded that IP6 promotes the resolution of exacerbated inflammatory responses in LPS‐stimulated BMDM.

## 4 DISCUSSION

There is an increasing need to develop plant‐based dietary supplements due to their biological potential to improve health and disease prevention. A growing body of evidence suggests that IP6’s antioxidant, anti‐inflammatory, and immune‐enhancing capabilities could increase bone density and reduce bone loss and hip fractures (del Mar Arriero et al., 2012a; Graf & Eaton, 1990; Graf et al., 1987; Kumar et al., 2004; López‐González et al., 2013; Zajdel et al., 2013; Zhang et al., 2005). Such drug‐like effects have been observed in the phosphate‐based drug Fosamax, a bisphosphonate medication used to treat inflammatory diseases such as osteoporosis and inflammation‐induced cancer (Johnell et al., 2003; Wysowski, 2009). However, due to the significant side effects of Fosamax, IP6 is considered to be a safer and more effective alternative medication because of its low toxicity and broader spectrum of bioactivities (Chakraborty et al., 2011; Kalam Shamsuddin & Bose, 2012).

**FIGURE 5:**
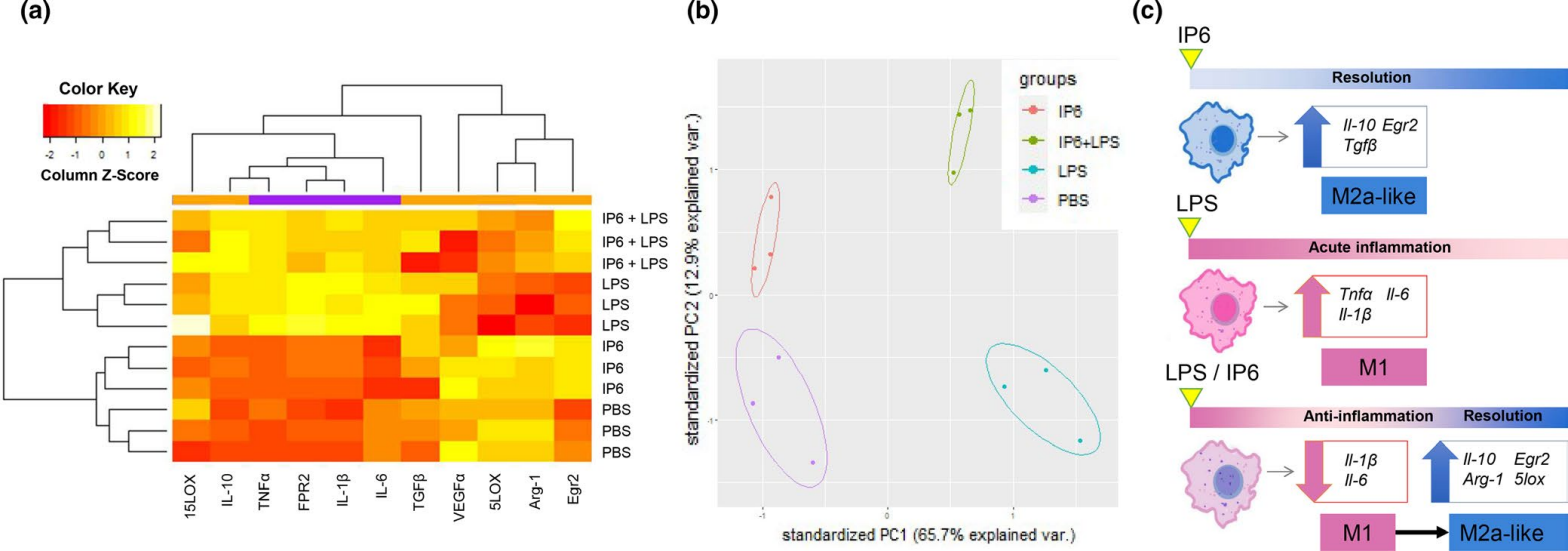
Induction of distinct gene expression patterns in BMDM by IP6. (a) Heat map showing the relative expression levels of macrophage polarization markers in BMDM treated with PBS, IP6, LPS, and IP6+LPS. Each gene's expression level is represented using an ordinal scale ranging from the minimum (yellow) to maximum (red). (b) Principal component analysis (PCA) score plot using 11 macrophage‐related markers shows separate clustering of PBS, IP6, LPS, and IP6 + LPS from each other. The variance between the principal component samples was 65.7% for PC1 and 12.9% for PC2. Colored dots represent triplicates, and colored ellipses represent the confidence interval for each group. (c) IP6 facilitates macrophage polarization toward the M2a‐like subtype. IP6 can also accelerate the transition process from the pro‐inflammatory phase toward resolving inflammation in LPS‐treated BMDM by altering the expression of genes associated with anti‐inflammatories and resolution of inflammation pathways

Consistent with those findings, our study suggested that IP6 is not harmful to BMDM because we did not observe any cytotoxic effects when treating the BMDM with 200 µM of IP6 in a Ki67 cell proliferation assay (Figure 1). Similar results were observed in J774A.1 cells (Figure S1). Moreover, our study demonstrated that IP6 has profound effects on regulating the gene expression of pro‐ and anti‐inflammatory responses and resolution of inflammation (Figures 2 and 4). Our study also suggested that IP6 significantly impacts macrophage polarization by altering gene expression in M0 BMDM (Figures 4 and 5). Macrophage polarization plays a vital role in regulating inflammation‐induced pathogenesis. Therefore, IP6 could potentially provide an environmental cue to modulate macrophage behavior beneficially (Figure 5c).

The intrinsic properties of macrophages allow them to easily adapt to surrounding stimuli and switch their cellular functions on or off by generating versatile signaling networks. For example, macrophages can respond to infection and secrete pro‐inflammatory cytokines. On the other hand, the same macrophages can be polarized to anti‐inflammatory phenotypes, which are opposed to pro‐inflammatory phenotypes, when they receive signals such as lipid mediators and apoptotic cell‐released molecules. These anti‐inflammatory macrophages secrete cytokines and growth factors, which are important for tissue healing (Fujiwara & Kobayashi, 2005; Stout et al., 2005). Polarized macrophages can be loosely categorized into classically activated pro‐inflammatory M1 macrophages and alternatively activated anti‐inflammatory M2 macrophages. However, growing evidence suggests that alternatively activated M2 macrophages can be further subdivided into M2a, M2b, M2c, and M2d subtypes based on environmental cues and the resultant transcriptional changes of distinct cytokine production. (Biswas et al., 2012; Liu et al., 2014; Wang et al., 2019).

Specifically, alternative M2a macrophages are known to be involved in tissue repair by generating IL‐10, TGFβ, ARG‐1, and EGR2 (Lu et al., 2013). Another subtype, the M2b macrophage, provides immunomodulation by secreting IL‐10, IL‐6, TNFα, and IL‐1β (Wang et al., 2019). For cytokine production, M2c macrophages are similar to M2a macrophages that secrete IL‐10, TGFβ, and ARG‐1, but without EGR2 production (Lu et al., 2013). The M2d subtype, also known as tumor‐associated macrophages (TAMs), secretes TNFα, IL‐6, IL‐10, TGFβ, and VEGFA to promote tumor progression (Chanmee et al., 2014). Our study revealed that IP6 is capable of polarizing macrophages to an M2a‐like subtype by increasing gene expression of *Arg‐1* and *Egr2* (Figures 2 and 4). Given that upregulation of *Vegfa* gene expression is found in TAM or M2d, we showed that treating BMDM with IP6 did not alter the gene expression of *Vegfa* (Figure 2). These findings have gone some way toward enhancing our understanding of the roles of IP6 in anti‐cancer activities. IP6 is less likely to promote cancer progression because of its ability to skew macrophage polarization toward an M2a‐like subtype instead of an M2d subtype that promotes angiogenesis in tumors via upregulation of *Vegfa* gene expression.

In addition to the polarization of macrophages to an M2a‐like subtype under the influence of IP6, we found that IP6 significantly alters the gene expression of *Fpr2* and *5lox*. These molecules are critical factors in the resolution of inflammation (Figure 4c) (Corminboeuf & Leroy, 2015, p. 2; Lasky et al., 2015). Recent studies suggest that activation of the FPR2 receptor by its ligands, such as specialized lipid mediators (SPMs), plays an essential role in maintaining homeostasis by returning excessive inflammation to a basal level. Moreover, loss of FPR2 in mice was associated with exacerbated inflammation in several diseases (Corminboeuf & Leroy, 2015; Tourki et al., 2020). These results suggest that activation or upregulation of FPR2 is critical for triggering the resolution of inflammatory pathways. It is also important to note that the gene expression of *Fpr2* is upregulated to counterbalance the exacerbated inflammatory responses caused by potent inducers of inflammation, such as LPS (Figure 4c). We found that the levels of *Fpr2* gene expression were downregulated in LPS + IP6 BMDM compared to LPS BMDM. The downregulation of overexpressed *Fpr2* in LPS + IP6 BMDM indicated that the inflammatory reaction in LPS + IP6 BMDM was less vigorous (Figure 4c). This suggested that IP6 is capable of reducing exacerbated LPS‐induced pro‐inflammatory responses. Upregulation of *5lox* further indicated that IP6, while reducing pro‐inflammatory responses, accelerates the transition process from the pro‐inflammatory phase toward resolving inflammation in LPS‐treated BMDM (Figure 4c).

We performed quantitative qPCR assays on cDNA prepared from BMDM and clustered BMDM on different treatments using default PCA methods. The results revealed that IP6 exhibited preferential induction of genes associated with anti‐inflammation and resolution of inflammation pathways, which organized IP6‐treated BMDM into a cluster that was transcriptionally distinct from other treatments (Figure 5a,b). Briefly, a comparison of gene expression in PBS, IP6, LPS, and IP6+LPS BMDM identified genes associated with pro‐inflammatory pathways; that is, M1 phenotypes were lower in IP6 than in PBS and lower in IP6 + LPS BMDM than in LPS. In contrast, transcripts associated with anti‐inflammatory responses and resolution of inflammation pathways, including *Il‐10*, *Arg‐1, Egr2, Tgfβ, Fpr2*, and *5lox*, were upregulated in IP6 compared to PBS and in IP6 + LPS BMDM compared to LPS (Figure 5a). In light of these findings, we conclude that IP6 might be used as a critical dietary component for regulating exacerbated inflammatory responses by influencing the expression of genes associated with anti‐inflammatory responses and resolution of inflammation pathways in macrophages (Figure 5c).

## 5 CONCLUSIONS

Our study results provide an insight into the role of IP6 as a dietary component to modulate the behavior of macrophages through alteration of gene expression involved in pathways of pro‐ and anti‐inflammatory responses, and resolution of inflammation pathways (Figure 5c). Our results also highlighted that dietary IP6 might offer cancer protection by skewing macrophage polarization toward the M2a‐like subtype. With these findings, IP6 may represent a healthy diet to shape macrophage functions. This effect would be expected to have a beneficial impact on diverse diseases are associated with uncontrolled inflammation.

## CONFLICT OF INTEREST

The authors declare no conflict of interest.

## **AUTHOR** **CONTRIBUTIONS**

Y.W. and C‐S.W. involved in conceptualization; Y.W., C‐H.Y., S‐K.C., Y‐C.Y., and C‐S.W. methodology; Y.W. and Y‐C.Y. involved in software; Y.W., C‐H.Y., S‐K.C., Y‐C.Y., and C‐S.W. involved in validation; Y.W., C‐H.Y., S‐K.C., Y‐C.Y., and C‐S.W. involved in formal analysis; C‐S.W. involved in resources, funding acquisition, project administration, and supervision; Y.W. and C‐S.W. data curation; Y.W. wrote the original draft; Y.W. and C‐S.W. wrote, reviewed, and edited, and involved in visualization;;.. All authors have read and agreed to the published version of the manuscript.

## AUTHOR CONTRIBUTION

**Yinshen Wee:** Conceptualization (equal); Data curation (equal); Formal analysis (equal); Investigation (equal); Methodology (equal); Validation (equal); Visualization (equal); Writing‐original draft (equal); Writing‐review & editing (equal). **Chieh‐Hsiang Yang:** Formal analysis (supporting); Methodology (supporting); Resources (supporting); Software (supporting). **Shau‐Kwaun Chen:** Formal analysis (supporting); Methodology (supporting); Software (supporting). **Yu‐Chun Yen:** Data curation (supporting); Formal analysis (supporting); Software (equal); Validation (supporting). **Ching‐Shuen Wang:** Conceptualization (equal); Data curation (equal); Formal analysis (equal); Funding acquisition (equal); Investigation (equal); Methodology (equal); Project administration (equal); Resources (equal); Supervision (equal); Validation (equal); Visualization (equal); Writing‐original draft (equal); Writing‐review & editing (equal).

## ETHICAL APPROVAL

This study was performed in accordance with the Institutional Animal Care and Use Committee (IACUC) of Taipei Medical University (TMU), Taipei, Taiwan. All animal experiments were approved by the IACUC (IACUC number: LAC‐2020–0331).

## Supporting information

Supplementary Material
