# Supplementary material for: Inositol hexaphosphate modulates the behavior of macrophages through alteration of gene expression involved in pathways of pro‐ and anti‐inflammatory responses, and resolution of inflammation pathways
Source: Food Sci Nutr. 2021 Apr 10;9(6):3240–9. doi: 10.1002/fsn3.2286 (PMC8194914; doi:10.1002/fsn3.2286)
Supplement: Supplementary file 1 — Supplementary Material [file FSN3-9-3240-s001.docx]

**Supplemental Data**


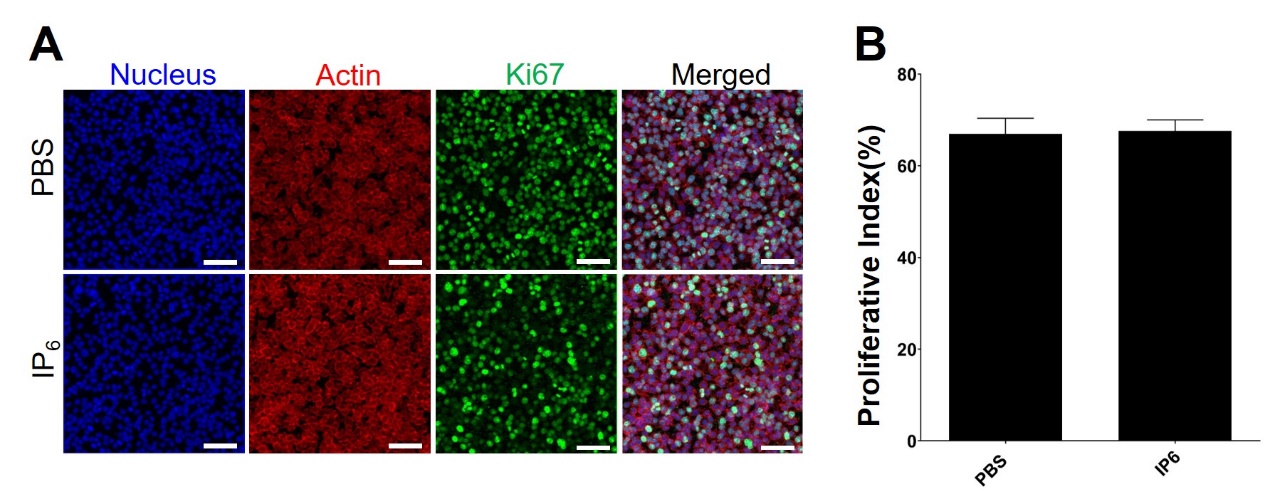


Figure S1. Treatment with IP6 does not affect the proliferation in J774A.1 cells. (A) Immunofluorescence staining of the proliferation marker Ki67 in each group: actin (red), Ki67 (green), and DNA (blue). The results from three independent experiments and representative images are shown. Scale bars = 50 μm. (B) Statistical analysis of Ki-67-positive cells in each group.

| **Gene** | **Forward (5’-3’)** | **Reverse (5’-3’)** |
| --- | --- | --- |
| m*Il-1β* | GCACTACAGGCTCCGAGATGAAC | TTGTCGTTGCTTGGTTCTCCTTGT |
| m*Tnfα* | CTGTAGCCCACGTCGTAGC | TTGAGATCCATGCCGTTG |
| m*Il-6* | TCCAGTTGCCTTCTTGGGAC | GTACTCCAGAAGACCAGAGG |
| m*Arg-1* | TTGGGTGGATGCTCACACTG | TTGCCCATGCAGATTCCC |
| m*Tgfβ* | CACCGGAGAGCCCTGGATA | TGTACAGCTGCCGCACACA |
| m*Il-10* | TGGCCCAGAAATCAAGGAGC | CAGCAGACTCAATACACACT |
| m*Egr2* | CCTTTGACCAGATGAACGGAGTG | CTGGTTTCTAGGTGCAGAGATGG |
| m*Vegfa* | CTGCTGTAACGATGAAGCCCTG | GCTGTAGGAAGCTCATCTCTCC |
| m*Fpr2* | GCCTTTTGGCTGGTTCCTGTGT | CAAATGCAGCGGTCCAAGGCAA |
| m*5lox* | TCTTCCTGGCACGACTTTGCTG | GCAGCCATTCAGGAACTGGTAG |
| m*15lox* | GACACTTGGTGGCTGAGGTCTT | TCTCTGAGATCAGGTCGCTCCT |
| m*β-actin* | GTAACAATGCCATGTTCAAT | CTCCATCGTGGGCCGCTCTAG |

**Table S1. qPCR primers used in this study.**
